# Ferroptosis as a Form of Cell Death—Medical Importance and Pharmacological Implications

**DOI:** 10.3390/ph18081183

**Published:** 2025-08-11

**Authors:** Blanka Kielan, Artur Pałasz, Krzysztof Krysta, Marek Krzystanek

**Affiliations:** 1 SUM Doctoral School, Medical University of Silesia in Katowice, 40-055 Katowice, Poland; 2 Department and Clinic of Rehabilitation Psychiatry, Faculty of Medical Sciences in Katowice, Medical University of Silesia in Katowice, 40-055 Katowice, Poland; ochojec@gmail.com (K.K.); m.krzystanek@sum.edu.pl (M.K.); 3 Department of Histology, Faculty of Medical Sciences in Katowice, Medical University of Silesia in Katowice, 40-055 Katowice, Poland; artiassone@gmail.com

**Keywords:** ferroptosis, lipid peroxidation, iron, cell death, neurodegenerative diseases, cancer diseases, autoimmune diseases, hemorrhages, ferroptosis inhibitors, ferroptosis inductors

## Abstract

**Background/Objectives:** Ferroptosis is a regulated form of cell death that occurs in the state of oxidative–antioxidative imbalance of an organism. The main components of ferroptosis are lipid peroxidation and iron accumulation. Cells experiencing ferroptosis show swelling, shrunken mitochondria with an abnormal structure, atrophic cristae, dense mitochondrial membranes, and ruptured outer membrane. Ferroptotic cells demonstrate a normal nucleus size without nuclear concentration, and neither condensation nor chromatin margination. Ferroptosis is regulated by multiple protein, genetic, and metabolic factors. The aim of this article is to present ferroptosis as a model of cell death occurring in various conditions and diseases. **Methods:** A literature search of PubMed, Web of Science was performed. Search terms included “ferroptosis”, “lipid peroxidation”, “iron”, and “cell death”. **Results:** Ferroptosis affects the onset, course, progression, and treatment of diseases, including neurodegenerative diseases, cancer diseases, autoimmune diseases, and hemorrhages. By using appropriate ferroptosis moderators, it is possible to influence the course of the disease in patients. **Conclusions:** By understanding the ferroptosis phenomenon well, it is possible to regulate its occurrence by considering the action of oxidative and antioxidant factors. A comprehensive understanding of ferroptosis and the factors regulating this process should be the goal in therapy for many diseases.

## 1. Introduction

Ferroptosis is a regulated form of cell death that occurs when the oxidative–antioxidant balance of the body is disturbed [1]. This happens when the functioning of the antioxidant system responsible for neutralizing lipid reactive oxygen species (ROS) is impaired, which results in the accumulation of lipid hydroperoxides in cellular membranes. Additionally, the intensified production of lipid ROS causes these effects [2].

An essential component of ferroptosis is lipid peroxidation [3]. Peroxidation includes free polyunsaturated fatty acids (PUFAs) and membrane phospholipids containing PUFAs (PUFA-PLs) [4]. PUFAs are incorporated into cellular membranes to create PUFA-PLs, which drives lipid peroxidation and ferroptosis. The whole process is controlled by enzymes discussed later in this article [5]. The most susceptible to oxidation are phosphatidylethanolamines (PEs) [4]. Mitochondria, structures highly vulnerable to lipid oxidation, are the main site of PE synthesis and endogenous ROS production [6]. The second important component of ferroptosis, which leads to oxidative damage, is iron accumulation [7]. Redundancy iron intensifies PUFA-PLs peroxidation via the production of ROS in Fenton reactions and by activating lipoxygenases or cytochrome P450 oxidoreductases [5].

Morphologically, ferroptotic cells are characterized by swelling, shrunken mitochondria with an abnormal structure, atrophic cristae, dense mitochondrial membranes, and ruptured outer membranes [7,8]. Ferroptotic cells demonstrate a normal nucleus size without nuclear concentration, and neither condensation nor chromatin margination [9]. Ferroptosis is activated and regulated by protein, genetic, and metabolic factors [10].

Ferroptosis, as a programmed form of cell death, may prevent diseases resulting from excessive cell proliferation, including cancer cells. Activation or inhibition of ferroptosis can modulate disease progression, as has been studied in animal models [11]. The role of ferroptosis in tumor suppression has been described, among others, in the case of ovarian cancer (Hong 2021) [12], bladder cancer (An 2023) [13], hepatocellular carcinoma (Huang 2023) [14], melanoma (Meng 2023) [15], and prostate cancer (Ghoochani 2021) [16].

Ferroptosis does not exhibit the same characteristics as other types of cell death. During ferroptosis there is no release of mitochondrial cytochrome c, activation of caspases, chromatin condensation, and nuclear membrane and cytoskeleton breakdown; these are characteristic symptoms of apoptosis [17,18]. Ferroptosis is mainly characterized by the destruction of the mitochondria. In autophagy, high molecular weight components of the cytoplasm are destroyed, and in necrosis, cytoplasm and organelle tumefaction occurs [19,20]. In the case of pyroptosis and necroptosis, there is a loss of cell membrane integrity, membrane perforation, cell swelling, and a release of inflammatory substances [9]. A recently discovered type of cell death, cuproptosis, is activated by copper ions and is related to protein lipoylation, mainly associated with the TCA cycle. This mechanism of cell death is also distinct from ferroptosis. The absence of these features led to the distinction of ferroptosis as a distinct form of cell death [21].

The aim of this article is to present ferroptosis as a model of cell death occurring in various conditions and diseases. The goal is to demonstrate the main mechanisms leading to ferroptosis because if these are well understood and regulated skillfully, the processes of various diseases can be influenced. This article looks to demonstrate the effects of ferroptosis inducers and inhibitors through the modulation of which cell death can be induced or inhibited.

## 2. Iron Homeostasis

### 2.1. Iron Circulation in the Body

The connection between iron and ferroptosis requires a review of iron homeostasis and an understanding of the mechanisms leading to an increase in iron levels in cells and the organism as a whole. Iron is a metallic trace element essential for the life of humans and all vertebrate species [22]. The element ingested with food in the form of ferric ions Fe^3+^ is solubilized and ionized in the stomach by the action of gastric acid [23]. Then, under the influence of reductase, the insoluble Fe^3+^ ions are reduced to more-easily absorbed ferrous Fe^2+^ ions, and, in this form, they are absorbed in the duodenum. Divalent cation transporters (DMT_1_, DCT_1_) and ferroportin 1 (FPN1) are responsible for transporting the absorbed iron ions from the surface of enterocytes to their cytoplasm and then to the bloodstream [24]. DMT1 enables the transport of Fe^2+^ ions into the cytoplasm of enterocytes, and ferroportin supports the export of iron from these cells to the portal vein circulation. The portal vein transports iron to the liver, where its largest storage is located [23]. Hepatocytes secrete the hormone hepcidin, which lowers blood iron levels by inhibiting iron export from enterocytes [25]. Hepcidin binds to ferroportin, causing conformational modification of ferroportin, and therefore directs ferroportin to the ubiquitination process. This leads to endocytosis of the attached molecules and their lysosomal degradation. Degradation of ferroportin blocks the release of iron into the plasma, thereby lowering blood iron levels [26].

### 2.2. The Hephaestin, Ceruloplasmin, and Transferrin Activity

The ferroxidase family proteins include hephaestin (HEPH) and ceruloplasmin (CP), which are responsible for the oxidation of Fe^2+^ to Fe^3+^. Hephaestin is located on the basement membrane of enterocytes, participating in intestinal iron absorption. Ceruloplasmin is found in blood plasma, helping iron emission from the liver and CNS. In the bloodstream, Fe^3+^ ions are captured by plasma transferrin (Tf), and then transported to the body’s cells [27]. When transferrin reaches the target cell, it binds to transferrin receptor 1 (TfR1), a membrane protein that transports iron into the intracellular compartment. The transferrin-Tfr1 complex is finally formed and internalized. The endosomal transferrin-Tfr1 complex is acidified, and in the low-pH environment of the lysosome, Fe^3+^ is reduced to Fe^2+^ by reductases [28,29]. On the endosome membrane there is a DMT1 transporter, which transports Fe^2+^ to the cell cytoplasm, where Fe^2+^ participates in important cellular processes. Serum transferrin is the main source of the required iron for cells [23].

### 2.3. Iron Circulation in a Cell

**Figure 1:**
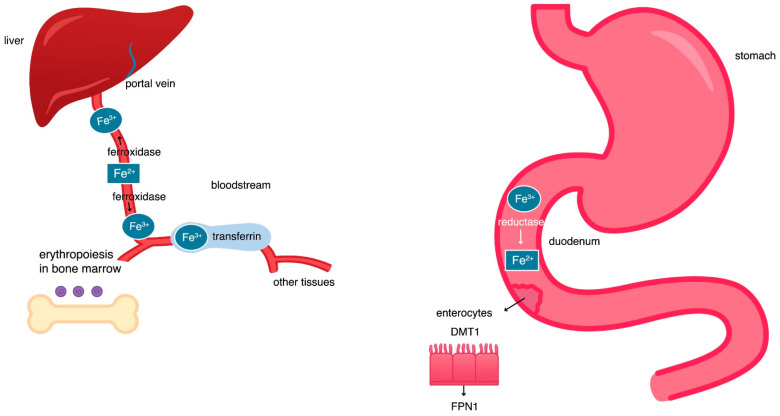
Iron circulation in the body [23,33,34,35].

**Figure 2:**
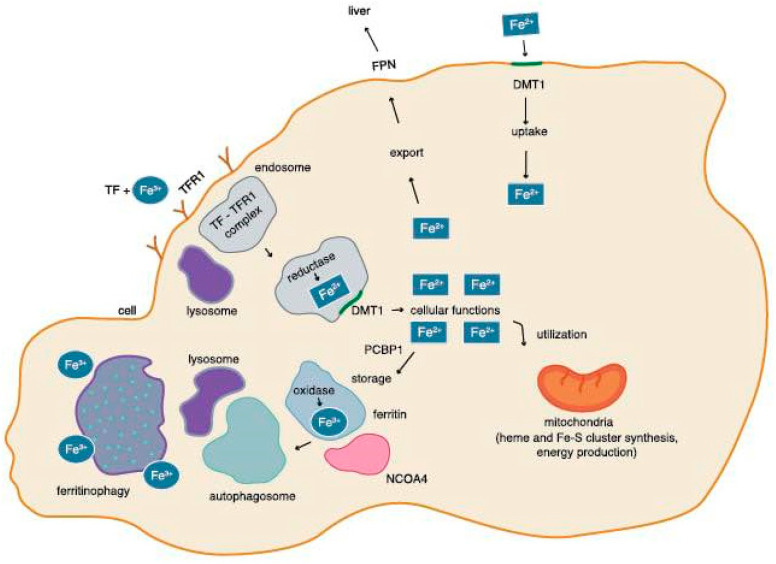
Iron circulation in a cell [23,33,34,35].

In cell cytosol, poly repeated cytidine (*rC*) binding protein 1 (PCBP1) directs ferrous Fe^2+^ ions to storage in the ferritin, and poly repeated cytidine (*rC*) binding protein 2 (PCBP2) directs ferrous ions into organelles or out of the cell. The ferritin core is made of ferritin heavy chains (FTH) and ferritin light chains (FTL) [30]. The H-subunit of ferritin exhibits ferroxidase activity and occurs mainly in the heart and kidney, whereas ferritin abundance in the L-subunit occurs mainly in the liver and spleen [31]. The heavy chain (H) of ferritin has oxidative properties that enable the oxidation of soluble Fe^2+^ transferred by PCBP1 to the insoluble Fe^3+^ form [30,31]. The release of insoluble iron from the ferritin core is mediated by the nuclear receptor coactivator 4 (NCOA4) during ferritinophagy. It is a physiological mechanism where NCOA4 binds to ferritin in the autophagosome; the autophagosome then fuses with the lysosome, which leads to the destruction of ferritin and the release of iron into the cytoplasm. The released iron is then used for cellular processes [32,33] (Figure 1 and Figure 2).

### 2.4. Regulation of the Level of Tron in a Cell by Ferritinophagy

The level of iron in the cell regulates the activity of the NCOA4 protein, which in turn participates in the process of ferritinophagy. High levels of iron in the cell triggers the interaction of NCOA4 with ubiquitin ligase. NCOA4 is then degraded by the ubiquitin proteasome system. As a result, the ferritinophagy process is inhibited. Ferritin does not release iron, which leads to a reduction in the level of iron in the cell. On the other hand, low iron levels in the cell determine the weakening of the interaction of NCOA4 and ubiquitin ligase, which increases the level of NCOA4 in the cell, and thus increases the level of iron through ferritinophagy [34]. The impairment of ferritinophagy disrupts iron homeostasis. If ferritinophagy does not occur, NCOA4 will not be able to degrade ferritin in the lysosome, so there will be excessive accumulation of both ferritin and iron inside the autophagosome. The release of iron into the cytoplasm will be inhibited and its intracellular concentration will decrease. On the contrary, enhancement of ferritinophagy promotes excessive release of iron into the cytoplasm, which drives processes leading to the synthesis of ROS, thus increasing the sensitivity of cells to ferroptosis [33,34].

### 2.5. Regulation of the Level of Iron in the Cell by IRP-1 and IRP-2

The level of ferritin translation is controlled by cytoplasmic proteins: iron regulatory protein-1 (IRP-1) and iron regulatory protein-2 (IRP-2). In the case of iron deficiency, IRP proteins bind to the iron-responsive element (IRE) sequence in the 5′-untranslated region (5′-UTR) of ferritin mRNA. This prevents ferritin translation, reduces its expression, and reduces the cell’s ability to store iron. At the same time, IRP-1 and IRP-2 bind to the IRE sequence located in the 3′-untranslated region (3′-UTR) of TfR1 and DMT1 mRNA. It commences upregulation of TfR1 and DMT1 in the cell, enhances iron uptake, and increases its level in the cell. In turn, in conditions of iron excess, its uptake is inhibited because the binding of IRPs to the IRE of TfR1 or DMT1 is blocked [22].

## 3. Excess Iron During Pathology Processes

### 3.1. The Fenton Reaction and the Haber–Weiss Reaction

Under physiological conditions, iron homeostasis processes are constantly monitored and function properly. Disturbances in iron homeostasis disrupt these mechanisms, leading to an iron overload within the cell.

During ferroptosis cells are overloaded with iron. The pool of non-transferrin-bound iron (*NTBI*) increases, playing a key role in pathological conditions associated with cell iron overload, which triggers the mechanism of synthesis of free oxygen radicals due to the reaction involving H_2_O_2_. Fe^2+^ is a catalyst in the Fenton reaction; the reaction of which is described by the equation:

Fe^2+^ + H_2_O_2_→Fe^3+^ + HO∙ + OH^−^,

An essential condition for the Fenton reaction, which produces ·OH, is the co-existence of Fe^2+^ and H_2_O_2_. The generation of ·OH radicals is strongly dependent on the pH of the solution. At acidic pH, H_2_O_2_ is stabilized as H_3_O_2_^+^ due to protonation. The H_3_O_2_^+^ ion is disproportionated into O_2_ and H_2_O, and Fe^2+^ oxidizes when the pH increases. Consequently, the Fenton reaction has an optimum pH between 3 and 4.

The regeneration of Fe^2+^ is possible using either oxygen species, the Haber–Weiss mechanism, or by organic reductants. The Haber–Weiss is a redox reaction that involves superoxide—O_2_^−^ and hydrogen peroxide—which forms a hydroxyl radical (·OH). The uncatalyzed reaction is very slow. In cells, the reaction is catalyzed by transition metals, such as Fe^3+^ and Cu^2+^, leading to a combined effect with the Fenton reaction [36,37,38,39].

O_2_^−^ + H_2_O_2_→O_2_ + HO∙ + OH^−^

O_2_^−^ + Fe^3+^→O_2_ + Fe^2+^

The change in the properties of ferrous and ferric iron is associated with the transport of electrons, making iron participate in key biochemical reactions of the cell, and in aerobic organisms enables the generation of ROS. Superoxide anion (O_2_^−^) and hydrogen peroxide (H_2_O_2_) do not oxidize important cellular macromolecules, but participate in the reaction to yield very reactive hydroxyl radicals (HO∙). The short-acting HO∙ radical reacts with other molecules, oxidizing the chemical groups which are closest to them, stripping them of their hydrogen atom, and adding hydroxyl in its place [40]. New radical molecules are then formed, which ultimately react to form peroxide radicals. Peroxyl radicals strip polyunsaturated fatty acids of their hydrogen atom, initiating membrane lipid peroxidation which occurs during ferroptosis [41].

### 3.2. Non-Transferrin-Bound Iron

NTBI, also known as labile plasma iron, involves a ferrous iron and a ligand-like carbonate, amino acid, or glutathione (GSH) [41]. NTBI occurs when transferrin is saturated. Iron coming from the absorption or degradation of hemoglobin cannot bind to transferrin (non-transferrin bound iron). It is in fact not labile, but rather redox-active iron [42], generated and accumulated in mitochondria [43]. In the cells, cytosolic ferrous iron is organized to form a labile iron pool (LIP) consisting of ferrous iron and used for, among other things, utilization in mitochondria [44]. LIP may originate from ferritinophagy, degraded hemoglobin, or the action of ferrireductase on ferric iron [41]. LIP is coordinated by GSH and associated with iron chaperones, PCBP1/2, which decides the ferrous iron transfer in a cell (for storage, export, utilization, etc.).

### 3.3. Antioxidant and Oxidative Factors

GSH action on Fe^2+^ aims to reduce Fe^2+^ reactivity and make iron utilization easier. In turn, free forms of ferrous iron, without chaperones, participate in the Fenton reaction, leading to hydroxyl radicals forming. Intracellular GSH decline is associated with LIP reactivity increase [44]. In the case of PCBP1 deficiency, unchaperoned iron increases, the redox-active LIP increases, and lipid peroxidation increases, leading to ferroptosis [44,45].

So, one of the goals of antioxidant therapy is, among others, to reduce the generation of HO∙ produced in the Fenton reaction [41]. The second conclusion is that intracellular transfer of ferrous iron and its concentration must be under tight control if the control mechanism is to function properly [42]. Iron contained in hemoglobin or ferritin, or iron bound to transferrin, is safe and essential for the body, but labile plasma or cellular iron and NTBI can lead to the formation of molecules that cause oxidative damage to cells [43].

### 3.4. Iron Regulatory Proteins

Binding iron to transferrin is necessary for the appropriate functioning of cells and it is possible because of the action of ferroxidases (ceruloplasmin, hephaestin). These components do not participate directly in the Fenton reaction but affect the iron metabolism in the body. The disfunction or mutation in the genes encoding these proteins can lead to the impairment of the effects of the importance of ferroxidases and iron collection in organs. Ceruloplasmin regulates the proper membrane distribution of ferroportin. As a result, a ceruloplasmin deficiency causes the disfunction of ferroportin. Lack of ferroportin activity prevents the export of iron from cells to the portal vein circulation, which leads to iron accumulation inside cells. Effectively, ferroxidase activity protects cells against an excessive ferrous iron level. Aceruloplasminemia, a disease with decreased or lack of ceruloplasmin ferroxidase activity, is defined as a neurodegenerative disorder due to the massive iron accumulation in the brain and other organs [38,39].

## 4. The Body’s Antioxidant System

### 4.1. Glutathione Peroxidase 4 as Component of Antioxidant System

Homeostasis of lipid oxidation and reduction reactions inside the cell depends on maintaining the balance between the oxidized and reduced forms of lipids, which allows for its proper functioning [3]. The purpose of the antioxidant system is to protect the body against excessive and uncontrolled cell oxidation. The protection of cell membranes is provided by glutathione peroxidase 4 (GPX4), which triggers the breaking-down of hydrogen peroxide into water and thus reduces the amount of oxidized lipids [46]. Additionally, GPX4 participates in the reduction of toxic lipid hydroperoxides to non-toxic lipid alcohols. GPX4 counteracts the effects of the Fenton reaction by neutralizing lipid peroxides and preventing oxidative damage in the cell [47].

### 4.2. Other Components of Antioxidant System

The regulation of mitochondrial ROS production is mediated by superoxide dismutase (SOD), which converts superoxide anions (O_2_^−^) into hydrogen peroxide (H_2_O_2_) and catalase (CAT). This reduces hydrogen peroxide (H_2_O_2_) to water (H_2_O) [48]. Thioredoxin reductase 1 (TXNRD1) reduces thioredoxins and is necessary for the proper functioning of antioxidant enzymes in the cell. This also provides protection against oxidative stress. TXNRD1 inhibitors are RAS-selective lethal 3 (RSL3) and Molecular Libraries 162 (ML162), known as ferroptosis triggers [49].

### 4.3. System Xc

Another element of the antioxidant system is also the Xc system, i.e., the amino acid antiporter, composed of the solute carrier family 7, member 11 (SLC7A11) and solute carrier family 3, member 2 (SLC3A2) subunits [50]. The heavy chain subunit SLC3A2 regulates the stability and proper positioning of SLC7A11 in the lipid bilayer. [51]. The SLC7A11 light chain subunit is a channel for the exchange of extracellular cystine and intracellular glutamate, which does not require the presence of sodium ions and is expressed in macrophages, astrocytes, and neurons [52]. By allowing cystine to enter the cell through the lipid bilayer, it facilitates the production of reduced glutathione–GSH–an endogenous compound that inhibits oxidation processes by the reduction of lipid peroxides, transforming itself into an oxidized form. The activity of GPX4, the enzyme that determines the antioxidant potential of cells, depends on the availability of the reduced form of glutathione; GSH is a co-factor of GPX4. The Xc system provides protection against the oxidative damage in cells caused by products of the Fenton reaction [50,53]. The activity of SLC7A11 is increased by the p53 tumor suppressor protein, which aims to reduce oxidative stress and prevents ferroptosis [3]. Some cells use a different pathway to produce cysteine from methionine, so in the case of Xc system failure, they are more resistant to ferroptosis-inducing Xc system inhibitors [8].

### 4.4. CoQ10

Ferroptosis suppressor protein 1 (FSP1) is a factor that prevents ferroptosis by the way of ubiquinone (CoQ10) [54]. CoQ10 partakes in carrying electrons in the electron transport chain in mitochondria [55]. FSP1 participates in the reduction of oxidized CoQ10 to ubiquinol through the help of NADH [56]. Ubiquinol catches lipid peroxyl radicals, and suppress lipid peroxidation [54]. In conclusion, through the glutathione (GSH) system, thioredoxin (TXN) system, and coenzyme Q10 (CoQ10) system, cells avoid undergoing ferroptosis [57].

## 5. Oxidative Effect

### 5.1. Oxidative Processes

Inhibition of the antioxidant action of the described mechanisms leads to uncontrolled cell oxidation and death [58]. Oxidative destruction involves nucleic acids, lipids, proteins, and generally wreaks havoc in the cell [41]. Blocking of the Xc system and GPX4 activity leads to reduced antioxidant potential, accumulation of lipid peroxides, and ferroptosis [10]. A decrease in the ratio of the reduced form of glutathione, GSH, to the oxidized form, GSSG, is a marker indicating the intensity of oxidative processes, and the depletion of GSH in the cell leads to its death [59].

### 5.2. ACSL4 and LPCAT3

Disturbance of the balance of redox processes occurs as a result of the accumulation of oxidized lipids in the plasma membrane. This is facilitated by the enzyme, acyl-coenzyme A synthetase long-chain family member 4 (ACSL4) [3], which participates in PUFA’s metabolism. The substrate of ACSL4 is arachidonic acid, an important PUFA. ACSL4 changes arachidonic acid into arachidonyl-CoA, which is then esterified into phospholipids. Lysophosphatidylcholine acyltransferase 3 (LPCAT3) and lipoxygenases (LOX) partake in the merging of arachidonyl-CoA to phospholipids on membranes [60]. Under the influence of these enzymes, PUFA undergoes changes to create PUFA-PLs, and then phospholipid hydroperoxides (PL-PUFA-OOH) [7]. The physical properties of cell membranes change by disrupting the transport and signaling functions of important membrane proteins by increasing the number of proteins that form pores in the cell membrane. This leads to increased permeability and the loss of the cell membrane’s integrity. An increase in the number of PUFA-rich phospholipids increases the number of folds in the cell membrane, which is the location where the peroxidation process occurs most strongly [6]. In this way, LPCAT3, also known as MBOAT5, promotes ferroptosis [61].

An important recent study by von Krusenstiern et al. indicates the endoplasmic reticulum (ER) membrane as a location of high PUFA concentration and the main target of lipid peroxidation in ferroptosis. The incorporation of lipid ROS into the ER membrane is enough to start the ferroptosis process. The researchers treated the cells with an inducer of ferroptosis (RSL3, FIN56, FIN02), and within 2 h they identified the peroxidation of ER. After 5 h, they noticed lipid peroxidation in the plasma membrane. When using erastin, ferroptotic death occurs more slowly [2].

## 6. Other Factors Regulating Ferroptosis

### 6.1. ACSL3 and Ferroptosis Inhibition

An interesting observation is that the induction of monounsaturated fatty acids (MUFA) leads to the reduced concentration of ROS in the membrane through the function of acyl-CoA synthetase long-chain family member 3 (ACSL3), which causes ferroptosis inhibition [3]. Attenuation of ferroptosis by the weakness of lipid peroxidation can also be triggered by ACSL4 deletion [3]. The phenomenon of ferroptosis was examined in the ovarian cancer stem cell line in which the ascl4 gene was suppressed. Cells in which the ascl4 gene was suppressed showed a weakened process of incorporation of PUFAs into membrane phospholipids, which ultimately inhibited lipid peroxidation [62].

### 6.2. MBOAT1, MBOAT 2, and Ferroptosis Inhibition

Membrane-bound O-acyltransferase domain-containing 1 and 2 (MBOAT1, MBOAT 2) are enzymes that participate in cellular phospholipid remodeling, independent of GPX4 but dependent on MUFA, cause the production of PL-containing MUFAs, and inhibit ferroptosis. Downregulation of MBOAT 1/2 increases PUFA-PLs, sensitizing cancer cells to ferroptosis, which can be useful in therapy for breast and prostate cancers [61,63].

### 6.3. Factors Leading to Induction of Ferroptosis

Ferroptosis induction is the induction of cell death, a therapeutic target in cancer where cell death and inhibition of tumor growth are desired. For this purpose, drugs already approved for use, experimental agents, cytokines that induce inflammation in the cell, and physical factors are being investigated [64]. Inducers of ferroptosis, among others, are erastin, RSL3, sorafenib, artesunate, sulfasalazine, statins, cytokines, and ionizing radiation [64]. Ionizing radiation inhibits the function of SLC7A11, increases activity of ACSL4, and induces ROS generation, leading to ferroptosis [5]. Cytokines like interferon-γ (IFN-γ) promote ferroptosis. IFN-γ increases the expression of ACSL4 in tumor cells, which influences the incorporation of arachidonic acid. This enhances the content of phospholipids associated with arachidonic acid, mostly phospholipids containing C16 and C18 acyl chains [60]. Moreover, IFN-γ downregulates SLC3A2 and SLC7A11 and lowers the antioxidant potential [3]. The properties of ferroptosis inducers are used to slow down tumor progression. Statins, drugs used for hypercholesterolemia, are used in the therapy of triple negative breast cancer by the induction of ferroptosis [65]. This subtype of breast cancer shows sensitivity to ferroptosis [66]. Erastin connects to the voltage-dependent anion channel (VDAC2/3) on the outer mitochondrial membrane, affecting ion transport and the membrane’s permeability [67]. Erastin enhances oxidative phosphorylation, reduces ATP synthesis via glycolysis, intensifies ROS formation, and induces mitophagy—mitochondria autophagy [68]. Erastin is an Xc system inhibitor, and it reduces GSH levels in the cell and leads to lipid peroxidation [69]. The effectiveness of erastin in inducing ferroptosis was tested in ectopic endometrial stromal cells, in which increased lipid ROS concentration was observed. The mitochondria were smaller, condensed, and shrunken, and the density of the membrane was increased. The cells showed excess iron and lower FPN activity, which accelerated ferroptosis. It would be worthwhile to conduct research on erastin as a therapeutic option for the treatment of endometriosis [70]. RSL3 and FIN56 reduced the catalytic properties of GPX4 [68]. In a mouse model study, RSL3 stifles inflammation caused by lipopolysaccharides (LPS) and activates nuclear factor erythroid 2-related factor 2 (Nrf2), which extinguishes proinflammatory cytokine production and leads to ferroptosis resistance. Thus, when RSL3 is used against diseases, the effects may be anti-inflammatory and, given the inhibition of GPX4, pro-ferroptotic [71]. According to the study by Cheff et al. (Cheff et al., 2023), TXNRD1 is a selenoprotein that protects against oxidation [49]. TXNRD1 is directly inhibited by both RSL3 and ML162, which is not the case with GPX4, as previously assumed [49]. However, source [68] claims that RSL3 itself reduces the catalytic properties of GPX4.

### 6.4. Factors Leading to Inhibition of Ferroptosis

Referring to ferroptosis reducers, Srs11-92 is an ferrostatin analog explored in mice models of a stroke induced by middle cerebral artery occlusion–reperfusion. Srs11-92 showed a protective effect in regard to nervous system damage, neuronal death, neuroinflammation, or oxidative processes by the way of Nrf2 [72]. Deferoxamine (DFX) as an iron chelator binds NTBI, LIP, and iron from hemosiderin and ferritin. Iron bound to transferrin or hemoglobin will not be taken up by DFX [73]. DFX inhibits ferroptosis in chondrocytes, increasing GPX4 and SLC7A11 activity whilst decreasing MDA (malondialdehyde, lipid peroxidation product) and Fe^2+^ collection in the cell, also enhancing Nrf2 expression [74]. Nrf2 enhances the antioxidant protection of cells by maintaining the balance of oxidation and reduction processes, protecting cells from ferroptosis [75]. DFX is able to cross the blood–brain barrier and binds iron into brain tissue, which may be useful in diseases associated with hemorrhages [76]. Lipoxygenases (LOX), enzymes that oxidize PUFA, lead to lipid hydroperoxide generation and the progression of inflammation. LOX inhibitors may be used as a method for the treatment of cancer and inflammatory diseases [77]. Zileuton, an arachidonate 5-lipoxygenase (ALOX5) inhibitor, is able to reduce the invasiveness and metastasis of pancreatic cancer, where increased ALOX5 activity has been observed [78]. Zileuton as an effective approach was also examined in cervical cancer [79]. Baicalein was tested in melanocytes, in which ferroptosis was induced. Baicalein increased GPX4 activity and reduced ferroptotic effects induced by RSL3 [80]. However, in a study involving colorectal cancer (CRC), baicalein was shown to be a ferroptosis trigger and cell death inducer in CRC [81]. Among the ferroptosis inhibitors, we can distinguish apomorphine, which has an activating effect on Nrf2 and is an agonist of dopaminergic receptors. Apomorphine restrains cell death induced by RSL3 in a mouse’s hepatoma cell line, and is able to trap radicals even better than Trolox or Fer-1 [82]. Inducers and inhibitors of ferroptosis are shown in Table 1.

## 7. Selected Clinical Implications

### 7.1. Cancer Diseases

Ferroptosis has been described as an ambiguous and sometimes unclear mechanism of action in carcinogenesis, since it can promote tumor growth by causing damage and inflammation to the tumor microenvironment. On the other hand, the induction of ferroptosis can inhibit tumor growth [64]. In cancer cells, an altered metabolism of arachidonic acid with the participation of LOX is observed. Therapies like LOX inhibitors may be used in some cancer cases [78].

Inducing cancer cell death may inhibit the development of some neoplasmic changes. Ferroptosis inducer, sorafenib, is a multikinase inhibitor used as a drug to inhibit cancer cell proliferation and angiogenesis [86]. In cancer treatments, a possible way to induce the death of cancer cells is to increase ROS production, especially HO∙ [41]. Sorafenib induces oxidative stress, increases ROS in mitochondria, and reduces retinoblastoma protein (RB) levels, thus inhibiting cancerous liver cells [87,88].

Artesunate (ART) is a derivative of artemisinin, has anti-inflammatory and anti-cancer properties, and is used in the treatment of malaria. In pancreatic ductal cancer cells, administration of artesunate causes its reaction with Fe^2+^ ions from the cytoplasmic iron pool. As a result, it leads to the production of ROS and then to ferroptosis, which means it has a cytotoxic effect on cancer cells [89].

In another case, a meta-analysis by Park et al. (Park 2021) [90] describes the association of high serum ferritin levels with an increased risk of pancreatic cancer. Ferritin is an iron storage protein; excess iron takes part in the Fenton reaction, which leads to the production of ROS and the induction of carcinogenesis. Ferritin degradation causes the release of a fraction of intracellular iron, the excess of which characterizes cells experiencing ferroptosis. The meta-analysis provides information on the variable expression of ferroptosis-related genes in pancreatic cancer cells like, among others, LPCAT3 and TP53, as well as increased expression of the MYC protooncogene. Moreover, MYC increases the expression of IRP2, which leads to an enhanced fraction of intracellular iron [90].

### 7.2. Subarachnoid Hemorrhage (SAH), Ischemic Stroke

As a result of stroke, due to the rupture of an aneurysm, blood leaks into the subarachnoid space. The morphological elements of the blood disintegrate, causing the cytotoxic, excitotoxic, and oxidative death of brain cells. The erythrocytes released into the subarachnoid space undergo hemolysis, which results in the release of large amounts of hemoglobin into the extracellular space; this initiates the development of an inflammatory response, leading to the weakening of the blood–brain barrier (BBB). At the same time, the weakened BBB will be more permeable, leading to increased inflammation. There is a temporary global ischemia of the brain area affected by the hemorrhage, causing symptoms of early brain injury (EBI) [91,92]. The impaired function of the BBB increases the free iron levels in the brain. Neurons take up iron from the blood. Excess iron causes intracellular iron accumulation, cell peroxidation, and oxidative stress [93]. In the mice model, ferroptosis was shown to occur during SAH. The use of ferroptosis inhibitors decreased the damage to the neurons and the activation of microglia [94]. Following subarachnoid hemorrhage, ferroptosis and high 15-lipoxygenase-1 (ALOX15) activity was observed in the glial cells and the endothelium during EBI [95]. Ferroptosis causes death of the nerve cells and glial cells [96]. Since SAH-induced ferroptosis is associated with the process of ferritinophagy, targeting autophagy inhibition could alleviate the course of EBI [97].

The occurrence of cells with iron accumulation in ischemic rat tissue near blood vessels might indicate the occurrence of ferroptosis. When ferroptosis occurs, inflammation occurs, which exacerbates cell death through the ferroptotic mechanism. Therefore, during brain tissue ischemia, ferroptosis and neuroinflammation coexist. During ischemic stroke, there is an increase in the production of arachidonic acid, increased expression of ACSL4, increased production of proinflammatory cytokines in brain tissue, neuroinflammation, enlargement of the infarct area, cell death by ferroptosis, and deterioration of the clinical condition. Suppressing the ACSL4 gene may be a way to inhibit lipid peroxidation, which is a determinant of ferroptosis [93].

### 7.3. Autoimmune Diseases

Li et al. (2021) [98] studied patients with systemic lupus erythematosus (SLE) in whom serum IgG antibodies and interferon-α blocked the expression of GPX4. They found the presence of morphological features of ferroptosis in neutrophil cells, similar to the changes observed in the group of healthy individuals treated with the ferroptosis inducer RSL-3. Patients with lupus have been shown to have higher levels of ROS in neutrophils and a reduced survival time. Ferroptosis has been identified as a major form of neutrophil death in SLE. The use of ferroptosis inhibitors (liproxstatin-1 and deferoxamine) prevented neutrophil ferroptosis. A mouse model was also investigated, in which GPX4 deficiency in neutrophils caused a lupus-like disease [98].

### 7.4. Neurodegenerative Diseases

Ferroptosis has been described as a factor accelerating the development of neurodegenerative diseases such as Parkinson’s disease (PD) [99] or Alzheimer’s disease (AD) [100]. β-amyloid precursor protein (APP) gene, which encodes the iron-responsive element sequence (IRE) located in the 5′-untranslated region of mRNA (5′-UTR), is associated with intracellular iron content. As intracellular iron levels increase, IRE expression in the 5′-UTR region of mRNA and APP translation are enhanced. The result is the overproduction of the APP and the accumulation of β-amyloid in cells, which is characteristic of AD [101,102]. To reduce the severity of amyloidosis, drugs targeting IRE in the APP 5′-UTR mRNA have been developed [103]

**Figure 3:**
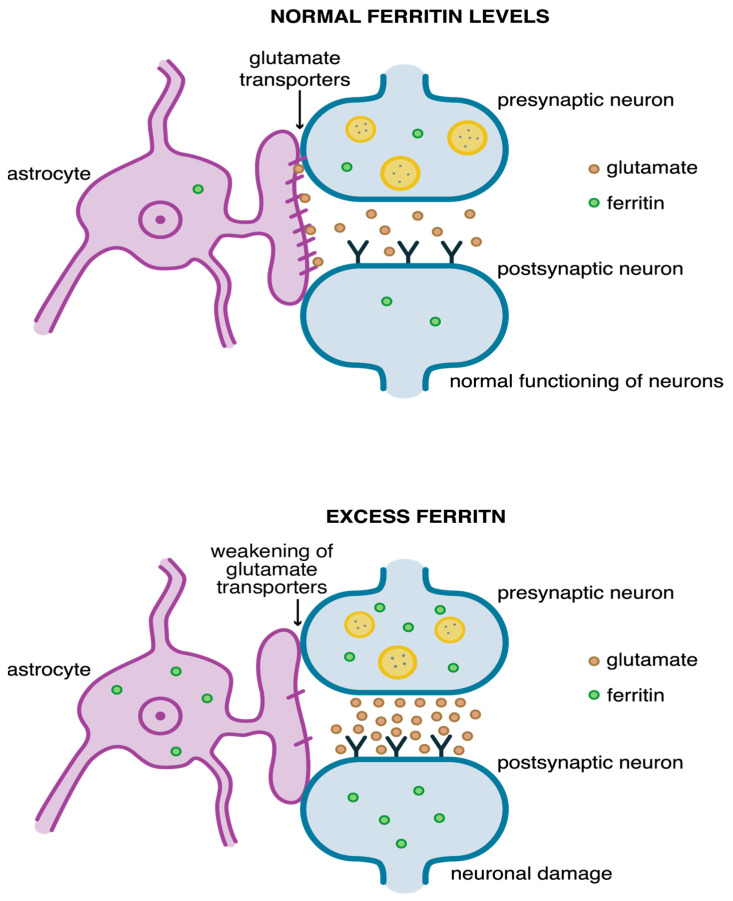
Impulse transmission leads to the removal of glutamate from the synapse via astrocytic glutamate transporter GLT-1 in the central nervous system. This allows it to maintain normal levels of glutamate (at the top). Excess ferritin levels inhibit glutamate uptake by synapses, which causes the overstimulation of neuronal receptors and their death, which happens during AD, PD, ischemia, etc., (at the bottom) [104,106].

Ferritin light chain (FTL) inhibits the uptake of glutamate from the extracellular space by “glial” GLT-1 transporters (glutamate transporter 1) located mainly on astrocytes [87,104]. Alekseenko et al. investigated the effect of ferritin on the uptake and release of glutamate and the formation of ROS in rat brain synaptosomes [104]. Synaptosomes are closed, plasma membrane-bound structures containing mitochondria, neuroplasm, and synaptic vesicles usually filled with neurotransmitter [105]. The study showed that high ferritin concentrations significantly reduced glutamate uptake by synapses. Inhibited glutamate uptake results in prolonged exposure of neurons to glutamate, which modifies synaptic transmission and leads to neuronal damage. Furthermore, ferritin induced ROS formation inside synaptosomes. Inhibition of glutamate uptake and the production of excess ROS in presynaptic neuronal terminals led to the activation of neurodegenerative processes in neurons (Alekseenko et al., 2008) [104]. The functioning of the glutaminergic synapse under normal conditions and under conditions of high ferritin concentrations is shown in Figure 3.

A similar effect is observed with excess iron in brain cells, which leads to brain damage, oxidative stress on nerve cells, and a predisposition for neurodegenerative diseases such as Parkinson’s disease [99].

In the course of PD α-synuclein (α-syn) aggregation occurring, amongst other processes, mitochondrial dysfunction and loss of lipid homeostasis takes place. Studies report a link between iron accumulation in the substantia nigra pars compacta (SNpc) in the brain of PD patients and the loss of dopaminergic neurons. Iron and α-syn occur in Lewy bodies in the midbrain of PD patients, and when iron connects to this protein, the conformation of α-syn changes and leads to the formation of cytotoxic α-syn aggregations [99].

In murine PD models, alpha lipoic acid (ALA), an iron chelator and antioxidant, reduced lipid peroxidation and the production of ROS, protected mitochondria, enhanced the expression of GPX4, and ultimately inhibited ferroptosis. It has been associated with alleviating motor deficits in PD [107].

### 7.5. Kidney Diseases

The study by Ye et al. (Ye 2022) [47] aimed to detect the presence of ferroptosis in a rat model of vascular calcification in chronic kidney disease (CKD). Thoracic aortic smooth muscle cells were isolated, in which the phenomenon of calcification was induced under conditions of increased calcium and phosphorus concentrations, which caused an increase in ROS levels. It was observed that the antioxidant mechanism was weakened and ferroptosis occurred. Ferrostatin-1 has been shown to be effective in reducing ROS levels and inhibiting lipid peroxidation and ferroptosis, as well as reducing intracellular calcium levels and limiting the development of arterial calcification. The study describes the antagonistic effect of erastin, an inducer of ferroptosis and an inhibitor of the Xc system [47].

Feng et al. investigated the phenomenon of ferroptosis in diabetic nephropathy in a mouse model of diabetes. One of the causes of diabetic nephropathy may be renal ischemia, which causes the release of hypoxia-inducible factor (HIF)-1α and a high level of heme oxygenase (HO)-1. Heme oxygenase triggers heme degradation and increased iron levels, which increases lipid peroxidation and ROS production. Diabetic mice showed increased expression of markers of renal tubular damage, including HIF-1α and HO-1, faster development of renal fibrosis, increased levels of iron and ROS in renal tubules, and decreased expression of antioxidant enzymes (SOD, GPX4, CAT). Administration of ferrostatin-1 to the research group of mice caused the opposite effects to those mentioned above, and thus inhibited the changes characteristic of ferroptosis [108]. Ferroptosis also occurs in the course of acute kidney injury (AKI), which influences kidney inflammation, and the main cause of ferroptosis in AKI is ACSL4. In turn, reduced ACSL4 activity protects against kidney damage [109].

### 7.6. Cardiovascular System

A randomized controlled trial by Zhang et al. evaluated the effectiveness of Qing-Xin-Jie-Yu Granule (QXJYG), a Chinese medicine method for the treatment of cardiovascular diseases, in regulating ferroptosis in atherosclerotic mice and RSL3-treated macrophages. Cells treated with QXJYG showed higher GPX4 activity, lower iron content, and reduced lipid peroxidation. QXJYG has been shown to attenuate ferroptosis in mice and inhibit the development of atherosclerosis [110].

### 7.7. General Anesthesia

Authors of another study associate ferroptosis with cognitive deficits and neurotoxicity which occurred after general anesthesia. The researchers examined the effects of anesthetic drugs on iron homeostasis, and it was found that the medications caused the accumulation of iron in hippocampal cells. Additionally, iron chelation with deferoxamine improved cognitive deficits and protected neurons from damage [111].

### 7.8. Intestinal Diseases

A meta-analysis by Hou et al. assess the impact of dexmedetomidine (DEX) on intestinal ischemia–reperfusion injury. The results show that DEX reduced the level of diamine oxidase, interleukin 1β, interleukin 6, tumor necrosis factor α, and malondialdehyde in animal models. DEX has a remedial effect on the disease; possible causes include its antioxidant, anti-inflammatory, and anti-ferroptosis effects. This substance stimulates the activity of SLC7A11 and GPX4, and also decreases iron levels [112] (Table 2).

## 8. Summary and Outlook

The aim of this article was to present ferroptosis as a model of the cell death occurring in various conditions and diseases. Ferroptosis influences the onset, course, progression, and treatment of the diseases, including neurodegenerative diseases, cancer diseases, autoimmune diseases, and hemorrhages. Triggers of ferroptosis could lead to the death of affected diseased cells, like using sorafenib in hepatocellular carcinoma cells [114], statins in triple negative breast cancer cells [65], and artesunate in pancreatic ductal cancer cells, ovarian cancer, and head and neck cancers [89]. As a result of the reduction of MBOAT1/2 expression, cells become sensitive to ferroptosis, which may be useful in combination with estrogen receptor (ER) and androgen receptor (AR) antagonists in the therapy of selected breast and prostate cancers [61]. Simultaneously, ferroptosis inhibitors, like Srs11-92, Fer-1 [72], liproxstatin-1 [94], or alpha lipoic acid [107], might have a protective effect on the nervous system during stroke or in neurological and neurodegenerative diseases.

A good understanding of the ferroptosis phenomenon allows us to regulate its occurrence by considering the action of oxidative and antioxidant factors. A thorough and comprehensive understanding of ferroptosis and the factors regulating this process can be the goal of therapy for many diseases. Extensive research shows the role of ferroptosis as a mechanism leading to disease, whilst at the same time being a consideration when developing new drugs and therapeutic strategies. The presented models of action of ferroptosis regulators in specific disease instances requires further research that will pave the way for their approval as drugs supporting the therapy of the specific diseases.

## 9. Materials and Methods

A literature search of PubMed, Scopus, and Web of Science was performed from June 2023 until April 2025. Search terms included “ferroptosis”, “lipid peroxidation”, “iron”, and “cell death”. Each database was searched individually to include unique indexing terms. Inclusion criteria involved the original research of articles, human studies, animal studies, articles in English, and studies describing the occurrence of ferroptosis and its association with various clinical conditions, as well as the use of ferroptosis inducers and inhibitors. Exclusion criteria involved conference abstracts, editorials, case reports, and studies not containing data on ferroptosis, its mechanisms, or its associations. The meta-analysis was not performed due to heterogeneity across studies. The initial search identified 280 articles. After the screening of titles and abstracts, 114 articles met the inclusion criteria. The other sources were excluded based on ineligible design and insufficient data.

## Figures and Tables

**Table 1:** Inducers and inhibitors of ferroptosis [8,49,83,84,85].

Inductors of Ferroptosis	Inhibitors of Ferroptosis
System Xc inhibitors Erastin Piperazine erastin (PE) Imidazole ketone erastin (IKE) Glutamate Sulfasalazine Sorafenib	Lipophilic antioxidants Ferrostatin-1 (Fer-1) SRS11-92 Liproxstatin-1 (Lip-1) Vitamin E Trolox CoQ10
HMG-CoA reductase Fluvastatin Lovastatin Simvastatin	Iron chelators Deferoxamine Deferiprone 2,2-bipyridyl
TXNRD1 inhibitors RAS selective lethal (RSL) 3 Molecular Libraries (ML)162	Lipoxygenase inhibitors Zileuton Baicalein
GPX4 inhibitors RSL3 Diphenyleneiodonium chloride (DPI) 7 DPI10 ML162 ML210 Altretamine Withaferin A (WA)	Others deuterated PUFAs glutaminolysis inhibitors cycloheximide dopamine β-mercaptoethanol selenium vildagliptin apomorphine
GPX4 degradation compounds Ferroptosis inducer 56 (FIN56) Caspase-independent lethal (CIL) 56	
GSH depletion compounds buthionine sulfoximine (BSO) acetaminophen	
Lipid peroxidation inducers FINO2 Artemisinin Others	
Carbon tetrachloride (CCl_4_)	

**Table 2:** Diseases in which ferroptotic cells have been identified and the efficacy of selected ferroptotic inhibitors.

Research	Disease	Ferroptotic Cells	Cell Features	Effective Inhibitors of Ferroptosis
Li et al., 2021 [98]	Systemic lupus erythematosus Lupus prone mice	Neutrophils	Mitochondrial vacuole creation, enlargement mitochondrial membrane density, atrophy of mitochondrial cristae	Liproxstatin-1 Deferoxamine
Ye et al., 2022 [47]	Chronic kidney disease	Vascular smooth muscle cells	High calcium and phosphate levels, high lipid ROS in cytoplasm	Ferrostatin-1
Feng et al., 2021 [108]	Diabetes	Renal tubule cells	High HIF-1α, HO-1, and iron levels, high lipid ROS, reduced SOD, GPX4, CAT	Ferrostatin-1
Zhang et al., 2023 [110]	Atherosclerotic cardiovascular diseases	Macrophage	Atherosclerotic cells: Swollen mitochondria, increased membrane density, atrophy of mitochondrial cristae	QXJYG–Qing-Xin-Jie-Yu Granule
Li et al., 2021 [70]	Endometriosis	Ectopic endometrium stromal cells	Shorter, condensed, shrunken mitochondria, increased membrane density	Ferrostatin-1, Liproxstatin-1, Deferoxamine
Yao et al., 2019 [113]	Spinal cord injury	Spinal cord cells	Shrunken mitochondria, rupture outer membrane	Deferoxamine

## Data Availability

Not applicable.
